# The Older They Are, the Less Successful They Become? Findings from the Georgia Centenarian Study

**DOI:** 10.1155/2012/695854

**Published:** 2012-07-29

**Authors:** Jinmyoung Cho, Peter Martin, Leonard W. Poon

**Affiliations:** ^1^Center for Applied Health Research, Scott and White Healthcare, Temple, TX 76508, USA; ^2^School of Rural Public Health, Texas A&M Health Science Center, College Station, TX 77845, USA; ^3^Human Development and Family Studies, Iowa State University, Ames, IA 50011, USA; ^4^Institute of Gerontology, University of Georgia, Athens, GA 30602, USA

## Abstract

This study examined whether oldest-old adults are successful agers. Three hundred and six octogenarians and centenarians of Phase III of the Georgia Centenarian Study participated in this study. A first model examined Rowe and Kahn's successful aging model (Rowe and Khan (1997 and 1998)) including the probability of disease, physical or cognitive capacity, and engagement with life. All three components were applied to assess how many oldest-old adults satisfied all three criteria. The result showed about 15% of octogenarians (15.1%), and none of centenarians satisfied all three components of successful aging. Consequently, a second alternative model focused on psychosocial aspects including three different components: subjective health, perceived economic status, and happiness. Different from Rowe and Kahn's successful aging model, a total of 62.3% of octogenarians and 47.5% of centenarians satisfied all three components of the alternative model of successful aging. The results suggest that additional criteria of successful aging should be considered thereby expanding the concepts and multidimensional aspects of successful aging among oldest-old adults.

## 1. Introduction

There is an oriental word—“*bullojangsaeng*”—which means physical immortality or external life in several Asian countries (e.g., Korea). The desire to live longer and healthy has been an aspiration of humankind for all ages and in all countries. The advancement of science has resulted in lower mortality. A consequence of lower mortality in the United States from 17.2 to 8.2 per 1,000 population and increases in life expectancy from 47 years in 1900 to 74 for men and 79 years for women [1] is that most people in the United States can expect to live longer. As the population of centenarians, another important segment of the older population, is expected to grow from 37,000 in 1990 to 850,000 in 2050 [2], it is important for oldest-old adults to understand successful aging when compared to relatively younger old adults. Even though increased longevity has been achieved, it is not clear whether increasing longevity is directly connected with successful aging. 

Since Rowe and Kahn [3, 4] proposed three indicators for successful aging (i.e., low probability of disease and disease-related disability, high cognitive and physical functional capacity, and active engagement with life), a number of studies have conceptualized successful aging indicators and examined older adults as successful agers based on the three criteria. However, many older adults have rarely satisfied these criteria because of the presence of disabilities and chronic diseases [5–8]. Moreover, oldest-old adults may easily fail to be categorized as successful agers when these criteria are applied. Kahn admitted that successful aging models should be complementary with other models [9], and successful aging model should encompass the criteria especially for oldest-old adults. For example, subjective health has been significantly correlated with functioning and mortality among oldest-old adults [10–14]. As subjective health has been generally viewed as a comprehensive single indicator of successful aging [15, 16], it is included as an alternative criterion instead of the physical health component of successful aging models in this study.

Several critical viewpoints of Rowe and Kahn's model have also suggested alternative indicators for successful aging models. George [17], for example, argued that the components of successful aging primarily focus on physical aspects and addressed an important question: “Is an older adult successfully aging if he/she is disability-free, physically and cognitive intact, and generally active, but rates the quality of life as poor or not good?” [17, page 322]. Although the majority of successful aging studies have included physical aspect as an essential factor for successful aging, psychological factors including emotional well-being have been identified as significant factors, especially in studies including very old adults [18, 19]. Thus, happiness, as a construct of life satisfaction or quality of life, in this study included “achievement of successful adaptation and expert survivorship in aging” ([13, page 3] and [20]).

Furthermore, Rowe and Kahn [3, 4] overlooked important aspects of aging such as financial resources, which directly or indirectly influence later life such as access to attain services for basic needs, physical health, living arrangement, and quality of life [21–25]. Since many oldest-old adults rely on family members' assistance, Social Security benefits, and the Medicaid program [26], perception or satisfaction of economic status may explain overall quality of life among older adults instead of objective income measures [27, 28].

The purpose of this study is to investigate Rowe and Kahn's [3, 4] “successful aging” model using data from the Georgia Centenarian Study. The overall objectives are to explore whether oldest-old adults are successful agers or not, to explore whether oldest-old adults are satisfied based on the criteria of successful aging, and to expand the psychological concept of successful aging among oldest-old adults. The following research questions are examined.

1. Will oldest-old adults satisfy the components of successful aging (i.e., physical health, cognitive/physical functioning, and engagement with life)?
2. Based on previous research, will oldest-old adults satisfy alternative criteria of successful aging (i.e., subjective health, financial resources, and happiness)?

## 2. Methods

### 2.1. Participants

As discussed in our previous work [29], the sampling frame of the Georgia Centenarian Study (GCS, Phase III) [30], which provides data for this study, had two components. The first one was to identify the proportion of all residents of skilled nursing facilities (SNFs) and personal care homes (PCHs) in a 44-county area in northern Georgia. Based on census proportions, the project recruited residents of SNFs and PCHs as well as community-dwelling residents. A second recruiting strategy was to use date-of-birth information in voter registration files. Based on these two components and five different characteristics (geographic, age, gender, ethnicity, and type of residence) a sample of octogenarians and centenarians was drawn for this study [30]. Information was collected through four sequential sessions, and information regarding resources and adaptation of older adults was the focus of this study.

Among 375 octogenarians and centenarians, three hundred and six participants (72 octogenarians and 234 centenarians) were left due to missing data and several proxies who had marginal mental status scores (proxy's MMSE < 23). The majority of octogenarians (69.4%) and centenarians (82.5%) were female. Over two-thirds of octogenarians (86.6%) lived in their own homes, whereas less than half of the centenarians (45.5%) lived in their own homes. As expected, most participants were widowed (centenarians: 86.3% and octogenarians: 53.7%). More octogenarians had an education beyond a high school degree than centenarians (octogenarians: 59.6% and centenarians: 40.4%). More octogenarians (86.1%) had high levels (MMSE ≥ 23) of cognition status functioning than centenarians (32.7%). In addition, in terms of the excluded 69 participants' information, they had similar characteristics when compared with samples used in this study. Most of them were female (73.9%), Caucasians (78.3%), widowed (77.9%), and over half of them (52.2%) lived in private home/apartments. There was no significant difference between the excluded and the remaining sample in mental status. A summary of demographic characteristics of the remaining sample of 306 can be found in Table 1.

### 2.2. Measures of Rowe and Kahn's Successful Aging Model

The criteria suggested by Rowe and Kahn [3, 4] were applied to oldest-old adults and included low probability of disease, physical or cognitive capacity, and engagement with life. Even though there is little agreement about the operationalization and definition of successful aging nor its measurement [31–34], each definition will follow the most often used operationalization as reviewed by Depp and Jeste [16]. Furthermore, the data used in this study were collected from proxy informants. It is not always easy nor feasible to obtain information from oldest-old adults. Many studies have shown a significant relationship between self and proxies or between self and physicians' reports [35] and that there was no potential bias such as disagreements between proxies and participants [36–40] or mean differences on mental health ratings between proxies and participants [41]. Therefore, we included data from proxy informants for several measures in this study.

#### 2.2.1. Low Probability of Disease

Rowe and Kahn [3, 4] defined “probability of disease” not only as the absence or presence of disease itself but also the absence, presence, or severity of risk factors for disease. Following this definition and based on Strawbridge et al. [8], absence of congestive heart failure, cancer, high blood pressure, Parkinson's disease, chronic pulmonary disease, and diabetes mellitus was used for low probability of disease. The reports were used based on centenarians' reports, proxy reports, medical reports, care facility's reports, or other available resources for best available information.

#### 2.2.2. High Cognitive/Physical Capacity

 As the second component of successful aging, physical/cognitive capacity was defined as potential for activities [3, 4]. *Physical capacity* was examined with instrumental activities of daily living (IADLs; seven items), and physical activities of daily living (PADLs; seven items) comprise the self-care capacity assessment [42] with coefficient *α* = .92 and *α* = .88, respectively. In addition, internal consistency of all 14 items was *α* = 0.94. All 14 items were scaled so that 2 = without help (e.g., can clean floors, etc.); 1 = with some help (e.g., can prepare some things but unable to cook full meals yourself); or 0 = completely unable to prepare any meals. Those who had no help with PADL were coded as high physical capacity ( = 1), and the remains were coded as low physical capacity ( = 0) [43]. *Cognitive capacity* was examined with the Mini-Mental Status Examination (MMSE) [44]. The MMSE is commonly used for evaluation of cognitive impairment. The MMSE is composed of five sections: orientation, registration, attention and calculation, recall, and language. The reliability is 0.98 for older adults, and concurrent validity with the Wechsler Adult Intelligence Scale was 0.78 in the original study [44]. The performance ranged from 0 to 30, and the thirty items yielded a reliability of *α* = 0.87 for this study. MMSE scores of 23 or higher were coded as high cognitive capacity ( = 1), and the remaining scores were coded as low cognitive capacity ( = 0) [45]. Taking high physical capacity and high cognitive capacity together was used as the second definition of Rowe and Kahn's model in this study [19].

#### 2.2.3. Active Engagement with Life

 Engagement with life, the final component of successful aging, was defined as interpersonal relations and productive activities [3, 4]. Active engagement with life was examined with two constructs, interpersonal relations and productive activity. For interpersonal relations, two questions of social support developed by Fillenbaum [42] were used: “How many people do you know well enough to visit with in his/her home or in their homes?” and “About how many times did you talk to someone—friends, relatives, or others on the telephone in the past week?” Those who had been spending time with family and/or friends at least once a week and having three or more people to visit were coded as active interpersonal relations ( = 1), and the remaining were coded as inactive relations ( = 0) [6]. For productive activity, experience of volunteer work was coded as “1” and the remaining were coded as “0” [46]. Both interpersonal relations and productive activity were considered together. 

### 2.3. Measures of Alternative Successful Aging Model

The criteria for alternative successful aging model follow the most frequently used operationalization as reviewed by Depp and Jeste [16] as well. 

#### 2.3.1. Subjective Health

Subjective health was used as a criterion instead of low probability of disease. Health status rated as either “good” or “excellent” was coded as good health ( = 1) and “fair” or “poor” was coded as poor health ( = 0) [47]. 

#### 2.3.2. Perceived Economic Status

Perceived economic status, which was neglected by Rowe and Kahn [3, 4], was included. Perceived economic status was measured with three items: the capacity to meet emergencies, to take care of needs, and to buy small luxuries [42]. Among the three items, those who were able to meet emergencies, to buy small luxuries, and to take care of needs fairly or very well were coded as better economic status ( = 1), and the remaining were coded as poor status ( = 0). 

#### 2.3.3. Happiness

 The last indicator for successful aging included psychological aspects in later life, happiness. The top third of the summary scores of three items (“I am just as happy now as when I was younger,” “My life could be happier than it is now” (reversed), “These are the best years of my life”) was used for happiness [19]. Those who had scores 1 to 3 on the summary scores of the three items were coded as happy ( = 1), and those who had scored −3 to 0 on the summary scores of the three items were coded as unhappy ( = 0). 

### 2.4. Data Analysis

Pearson's Chi-squared tests were performed to identify study participants to achieve the criteria of original successful aging model and the alternative successful aging model. Data were analyzed using the SPSS Statistical Software Package (version 19.0; SPSS).

## 3. Results

The results are separated into two sections. First, Rowe and Kahn's successful aging model was examined with octogenarians and centenarians. Three components were investigated separately and aggregately by age groups. Second, an alternative successful aging model with three criteria (i.e., subjective health, perceived economic status, and happiness) was analyzed in the same manner as in the first section.

### 3.1. Oldest-Old Adults and Rowe and Kahn's Successful Aging Model

Each component was applied to octogenarians and centenarians, and how many octogenarians and centenarians satisfied each component was investigated. For “low probability of disease,” 28.8% of octogenarians and 29.5% of centenarians satisfied this criterion. Over half of octogenarians (58%) and 4.4% of centenarians satisfied the physical and cognitive capacity criteria. There was a significant difference in association between age and physical/cognitive capacity among octogenarians and centenarians, *χ* ^2^(1, *N* = 295) = 107.67, *P* < 0.001. For engagement with life, 63.5% of octogenarians and 57.5% of centenarians met the third criterion (Table 2). Therefore, we can argue that physical and cognitive capacities are critical factors distinguishing octogenarians and centenarians.

**Figure 1:**
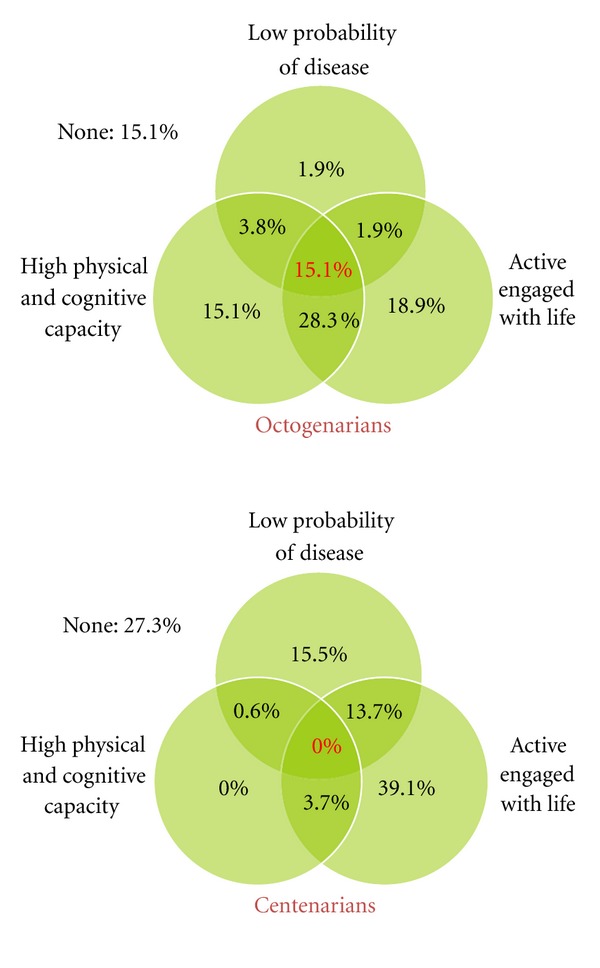
Drawings of age group comparisons for original successful aging model between octogenarians and centenarians. Numbers represent the proportion of each age group satisfied with components of the successful aging model.

Next, all three components were applied to investigate how many octogenarians and centenarians satisfied all criteria.  Figure 1 shows the combined proportion of participants, octogenarians, and centenarians, who satisfied three components of successful aging. Over 15% of octogenarians (15.1%) and none of centenarians satisfied all three components of successful aging. In addition, 15.1% of octogenarians and 27.3% of centenarians did not achieve any of the three components of successful aging. One-third (34%) of octogenarians and 18% of centenarians met two of the three components. Over one-third of octogenarians (35.9%) and 54.6% of the centenarians achieved only one component of successful aging. Specifically, it might be worthy to note that although centenarians had a high probability of disease and lower potential capacities, 39.1% of them had a high level of life engagement. Therefore, based on Rowe and Kahn's [3, 4] criteria of low probability of disease, high physical and cognitive capacity, and engaged lifestyle, we could argue that living longer does not necessarily imply successful aging.

### 3.2. Oldest-Old Adults and Alternative Successful Aging Model

However, are these three criteria the only viable aspects of successful aging among oldest-old adults? The three criteria have been commonly used to examine successful aging but, as Kahn [9] suggested, the successful aging model should be complemented with other models. Hence, it might also be necessary to expand the definition of successful aging. An alternative model for successful aging with three criteria (i.e., subjective health, perceived economic status, and happiness) is explored in this section. 

Each component was applied to octogenarians and centenarians, and how many octogenarians and centenarians were satisfied with each component was investigated. For “subjective health,” 77.5% of octogenarians and 73.0% of centenarians satisfied this criterion. Over three quarters of octogenarians (78.8%) and 61.8% of centenarians satisfied the perceived economic status criterion. There was a significant difference in association between age and perceived economic status among octogenarians and centenarians, *χ* ^2^(1, *N* = 286) = 6.49, *P* < 0.05. This indicates a larger proportion of octogenarians satisfied the perceived economic status criterion compared to centenarians. For happiness, 89.8% of octogenarians and 89.7% of centenarians met the third criterion (Table 3). Therefore, compared to Rowe and Kahn's [3, 4] criteria, more oldest-old adults satisfied these alternative aspects of successful aging criteria.

**Figure 2:**
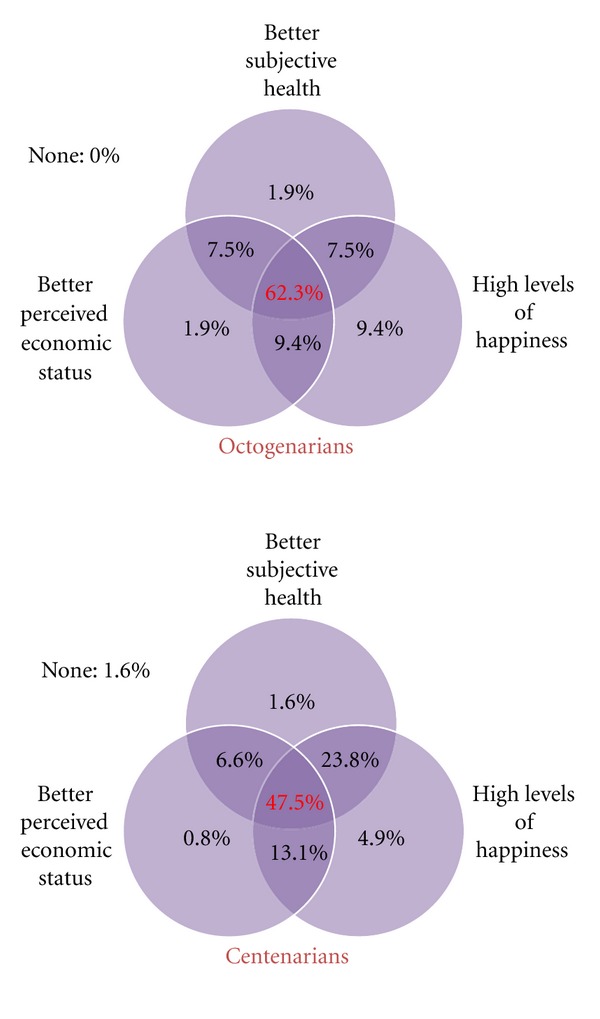
Drawings of age group comparisons for alternative successful aging model between octogenarians and centenarians. Numbers represent the proportion of each age group satisfied with components of the alternative successful aging model.

As was done with Rowe and Kahn's [3, 4] successful aging criteria, all three components for the alternative model were applied to investigate how many octogenarians and centenarians satisfied all criteria.  Figure 2 shows the combined proportion of participants, octogenarians and centenarians, who satisfied the three components of successful aging. A total of 62.3% of octogenarians and 47.5% of centenarians satisfied all three components of the alternative model of successful aging. Less than 25% of octogenarians (24.4%) and less than half of centenarians (43.5%) met two of the three components. Over 10% of octogenarians (13.2%) and 7.3% of centenarians achieved only one component of successful aging. In addition, no octogenarian and only 1.6% of centenarians did not achieve any of the three components of the alternative successful aging model.

## 4. Discussion

Rowe and Kahn's successful aging model was applied to oldest-old adults in this study. The components of the successful aging model included low probability of disease, high cognitive/physical functional capacity, and active engagement in life. The first research question was whether oldest-old adults would satisfy all three components of the successful aging model. Only 15.1% of octogenarians and none of the centenarians satisfied all three components. There is one obvious reason why oldest-old adults did not maintain all three components of the successful aging model. The participants used in the Rowe and Kahn study were 70 to 79 years old [3, 4]. The participants in this study were over 80 years of age, and most of them were centenarians. Physical health, which is a critical factor in Rowe and Kahn's aging model defined as the absence of disease and disability, shows dramatic decline after the age of 80 [48]. This might be the primary reason that our participants, especially centenarians, did not satisfy the criteria of Rowe and Kahn's successful aging model. 

Because this research question was not supportive of Rowe and Kahn's model with oldest-old adults, an alternative successful aging model was suggested (Research Question 2). The reason to suggest an alternative successful aging model is that successful aging should not be limited to a few concepts and variables. Kahn [9] agreed that the successful aging model should be complemented by other models or have a broader definition to adjust for the limitation that only a significant number of people could reach advanced age free of age-associated disease and without appreciable functional deterioration [8]. Consistent with other centenarian studies, this study confirmed the fact that it is naturally difficult, if not impossible, to reach advanced age free of diseases and disability [49–52]. 

The alternative successful aging model provided us with a different picture of successful aging for advanced old age. Instead of low probability of disease, high cognitive/physical functional capacity, and active engagement in life, subjective health, perceived economic status, and happiness were included. The results showed that 62.3% of octogenarians and 47.5% of centenarians met the criteria of the alternative successful aging model. Perhaps the results can best be interpreted with the compensatory mechanism or resilience. In other words, although physiological change or functional deterioration is closely associated with increasing age, psychological and social aspects of aging may not have positive relationships with physiological changes across the life span [52]. Therefore, certain psychological or social mechanisms such as happiness, positive affect, and social ties may compensate for physiological decline and allow some older adults to age successfully [52]. Thus, this alternative aspect of the successful aging model may contribute to the multidimensional successful aging construct and help older adults achieve successful aging even under conditions of physical health limitations and disabilities.

Even though this study provides an innovative perspective on successful aging, it is important for researchers to pay attention to a couple of limitations. First, the sample of this study was from only one geographic area of the United States. Other oldest-old adults in different regions might present different patterns of successful aging. In addition, there were some discrepancies between the proportions of sex and race in this study and national statistics. The 2010 census data, for instance, indicated that there were 36.3% male and 63.7% female people among the population of 80 years old and older [53], whereas our data included 79.4% of women and 20.6% men. Homogeneity may have limited the interpretation and application of these results to larger populations of oldest-old adults across the country. Second, we should consider different time points to assess whether oldest-old adults are successful agers or not. In other words, we need to consider survivorship effects when interpreting the results. Baltes and Smith [54] suggested that studies focusing on very old age pay attention to survival and mortality [55, 56]. For example, we can assume that survivors into very late life at some point in their lives had better scores in some domains such as health, intelligence, education, and psychological aspects than their counterparts who died prematurely or were close to death when being assessed [54]. Therefore, even though none of the centenarians satisfied the criteria of the successful aging model compared to 15.1% of octogenarians, centenarians might have at some point in their lives been functioning better on the three criteria of successful aging than those who had died earlier. Thus, we should differentiate age differences from selective survivorship in terms of considering longevity and successful aging. Finally, it is noted that the original successful aging model used objective measures, but alternative criteria of this study were not objective measures. A number of studies have shown discrepancies between self-rated-successful-agers and successful-agers-based Rowe and Kahn's criteria (e.g., [8, 57]). However, as Kahn [9] suggested, the purpose of this study was to contribute diverse aspects to a successful aging model among oldest-old adults rather than comparing two equivalent models. 

Despite these limitations, this study revealed interesting insight into the successful aging model of oldest-old adults. This information has numerous implications for gerontologists and practitioners. In particular, this investigation suggests that the successful aging model may not apply to oldest-old adults. Researchers and practitioners should consider many different factors for successful aging. Future research on oldest-old adults should use sequential designs when assessing successful aging. For example, investigators may want to apply the successful aging model to different cohort groups for several years. Based on this sequential design, the investigators may be able to explore the simultaneous examination of time and age effects for successful aging. 

It may be difficult to achieve successful aging in extremely late life. There is still no agreement on the definition of successful aging, and future work needs to expand the criteria for successful aging. In addition, more work needs to be done to examine predictors of successful aging as parts of developmental processes. Future work will contribute to the study of successful aging and help older adults achieve successful aging for as long as possible with a systematic approach to consider the past and present life and with a holistic view to understand age-related changes and challenges.

## Figures and Tables

**Table 1:** Summary of semographic characteristics.

Demographic characteristics	Octogenarians (*n* = 72)		Centenarians (*n* = 234)		*χ* ^2^
	*n*	%	*n*	%
Gender					5.72^∗^
Female	50	69.4	193	82.5	
Male	22	30.6	41	17.5	
Type of residence					35.52^∗∗∗^
Private home/Apartment	58	86.6	97	45.5	
Personal care (Assisted Living)	1	1.5	41	19.2	
Nursing home	8	11.9	75	35.2	
Ethnicity					2.02
White/Caucasian	61	84.7	179	76.5	
Black/African American	11	15.3	55	23.5	
Education					22.58^∗∗^
0–4 years	1	1.5	11	4.9	
5–8 years	2	3.0	53	23.8	
Some high school	6	9.0	26	11.7	
High school diploma	18	26.9	43	19.3	
Trade school or vocational degree	8	11.9	28	12.6	
Some college	9	13.4	22	9.9	
College degree	13	19.4	19	8.5	
Graduate degree	10	14.9	21	9.4	
Marital status					54.15^∗∗∗^
Never married	1	1.5	9	4.3	
Married	26	38.8	10	4.7	
Widowed	36	53.7	182	86.3	
Divorced	4	6.0	9	4.3	
Separated	0	0.0	1	0.5	
Cognitive status					63.59^∗∗∗^
Low (MMSE ≤ 17)	9	12.5	112	48.3	
Mid (18 ≤ MMSE ≤ 22)	1	1.4	44	19.0	
High (MMSE ≥ 23)	62	86.1	76	32.7	

*P < 0.05. ^∗∗^ *P* < 0.01. ^∗∗∗^ *P* < 0.001.

**Table 2:** Proportion of successful aging criteria.

	Octogenarians	Centenarians	*χ* ^2^
Low probability of disease	28.8%	29.5%	.01
High physical and cognitive capacity	58.0%	4.4%	107.67^∗∗∗^
Active engagement with life	63.5%	57.5%	.72

^∗∗∗^ *P* < 0.001.

**Table 3:** Proportion of alternative successful aging criteria.

	Octogenarians	Centenarians	*χ* ^2^
Better subjective health	77.5%	73.0%	.57
Better perceived economic status	78.8%	61.8%	6.49^∗^
High level of happiness	89.8%	89.7%	.00

^∗^ *P* < 0.05.
